# Retraction: The Effects of Physical Exercise on Cognition: How Heart Rate Variability Can Predict Cognitive Performances

**DOI:** 10.3389/fnhum.2020.628402

**Published:** 2021-01-26

**Authors:** Frontiers Editorial Office

The journal retracts the 31 August 2020 article cited above.

Following the publication of the article, the authors have identified a technical error in the execution of the tests included. The authors have corrected this error and re-conducted the analyses. However, the pattern of findings reported in the paper are no longer supported by the analyses.

The authors concur with the retraction and sincerely regret any inconvenience this may have caused to the reviewers, editors, and readers of Frontiers in Human Neuroscience.

